# Differential response to bacteria, and TOLLIP expression, in the human respiratory tract

**DOI:** 10.1136/bmjresp-2014-000046

**Published:** 2014-09-11

**Authors:** Olga Lucia Moncayo-Nieto, Thomas S Wilkinson, Mairi Brittan, Brian J McHugh, Richard O Jones, Andrew Conway Morris, William S Walker, Donald J Davidson, A John Simpson

**Affiliations:** 1 University of Edinburgh/MRC Centre for Inflammation Research, University of Edinburgh, Edinburgh, UK; 2 Centre for Infectious Diseases, The Chancellor's Building, University of Edinburgh, Edinburgh, UK; 3 Institute of Life Science, Medical Microbiology and Infectious Disease, Swansea University, Swansea, UK; 4 Department of Anaesthesia, University of Cambridge, Cambridge Biomedical Campus, Hills Road, Cambridge, UK; 5 Department of Cardiothoracic Surgery, Royal Infirmary of Edinburgh, Edinburgh, UK; 6 Institute of Cellular Medicine, Newcastle University, Newcastle upon Tyne, UK

**Keywords:** Innate Immunity

## Abstract

**Objectives:**

The observation that pathogenic bacteria are commonly tolerated in the human nose, yet drive florid inflammation in the lung, is poorly understood, partly due to limited availability of primary human cells from each location. We compared responses to bacterial virulence factors in primary human nasal and alveolar cells, and characterised the distribution of Toll-interacting protein (TOLLIP; an inhibitor of Toll-like receptor (TLR) signalling) in the human respiratory tract.

**Methods:**

Primary cells were isolated from nasal brushings and lung tissue taken from patients undergoing pulmonary resection. Cells were exposed to lipopolysaccharide, lipoteichoic acid, peptidoglycan, CpG-C DNA or tumour necrosis factor (TNF). Cytokines were measured in cell supernatants. TOLLIP was characterised using quantitative real-time PCR and immunofluorescence.

**Results:**

In primary alveolar, but not primary nasal, cells peptidoglycan significantly increased secretion of interleukin (IL)-1β, IL-6, IL-8, IL-10 and TNF. TLR2 expression was significantly higher in alveolar cells and correlated with IL-8 production. TOLLIP expression was significantly greater in nasal cells.

**Conclusion:**

In conclusion, primary human alveolar epithelial cells are significantly more responsive to peptidoglycan than primary nasal epithelial cells. This may partly be explained by differential TLR2 expression. TOLLIP is expressed widely in the human respiratory tract, and may contribute to the regulation of inflammatory responses.

**Key messages**

- Peptidoglycan exerts a significant proinflammatory cytokine response in primary human alveolar epithelium but not in primary human nasal epithelium.
- The Toll-like receptor regulator Toll-interacting protein is widely expressed in the human respiratory tract.

## Introduction

Hospital-acquired infections (HAIs) are common and associated with significant morbidity and mortality.^1^ Pneumonia is associated with the highest mortality among the HAIs.^1^ ^2^ The pathogenesis of hospital-acquired pneumonia is thought to involve recurrent microaspiration of mircoorganisms which have asymptomatically colonised the patient's oropharynx/nasopharynx during the course of hospital admission.^2^

Why the nasal epithelium should tolerate these microorganisms well, while the alveolar epithelium mounts such a florid inflammatory response, remains poorly understood. A better understanding of this paradox has been hampered by difficulties in accessing primary cells from the human nose and alveoli.

We therefore sought to characterise the effects of key virulence factors from *Staphylococcus aureus* and *Pseudomonas aeruginosa* (recognised as key pathogens in nosocomial pneumonia)^2^ on human primary nasal and alveolar epithelial cells. An additional aim was to determine whether Toll-interacting protein (TOLLIP, an endogenous inhibitor of Toll-like receptor (TLR) signalling)^3^ ^4^ was expressed in the human respiratory tract and, if so, whether there was differential expression in nasal and alveolar epithelium. This protein has been implicated as a key regulator of inflammatory responses in the large intestine, contributing to the dampening of TLR responses to microbe-associated molecular patterns derived from the extensive community of commensal organisms.^5^ ^6^ However, remarkably little is known about TOLLIP expression in the human respiratory tract.

The primary hypothesis for this study was that primary alveolar cells would mount a brisk response to inflammatory stimuli, associated with minimal or absent TOLLIP expression, whereas primary nasal cells would exhibit a blunted response to inflammatory stimuli, associated with abundant TOLLIP expression.

## Methods

### Derivation of cells

Primary human nasal epithelial cells, bronchial epithelial cells and type II alveolar epithelial cells were obtained from patients undergoing elective pneumonectomy or lobectomy for cancer. Methods for obtaining and culturing the nasal and alveolar cells have been described elsewhere.^7^ ^8^ Bronchial epithelial cells were obtained using a cytology brush passed through an endotracheal tube during the surgical procedure. Cells were seeded onto plates coated with type I rat tail collagen (Sigma-Aldrich, St Louis, Missouri, USA) and allowed to achieve confluence. Cells were studied at passage 2. Informed written consent was provided by all participants providing primary cells.

The human colonic carcinoma cell line T84 and the human nasal carcinoma cell line RPMI 2650 were from LGC Promochem (Manassas, Virginia, USA; ATCC numbers CCL-248 and CCL-30 respectively). A549 cells (derived from a human alveolar cell carcinoma) were available in-house.

### Cell stimulation experiments

Confluent cells were treated with 100 ng/mL of ultrapure lipopolysaccharide (LPS) derived from *P. aeruginosa* strain PA01 (a gift from Professor Ian Poxton, University of Edinburgh), 10 μg/mL of *S. aureus* peptidoglycan (PGN; Fluka, Sigma-Aldrich), 10 μg/mL of *S. aureus* lipoteichoic acid (LTA; Sigma-Aldrich), 10 ng/mL of recombinant human tumour necrosis factor (TNF; R&D Systems, Minneapolis, USA), 1 μΜ CpG-C DNA (ODN 2395; HyCult Biotechnology b.v., Uden, the Netherlands) or medium alone (all final concentrations). Cells were incubated for 24 h at 37°C and supernatants were removed and stored at −80°C until estimation of interleukin (IL)-1β, IL-6, IL-8, IL-10, IL-12p70 and TNF assayed using the BD Cytometric Bead Array (CBA) Human Inflammatory Cytokine kit (BD Biosciences), with analysis performed using a BD FACSArray Bioanalyzer System.

### RNA extraction, reverse transcriptase PCR and real-time quantitative PCR

Total RNA was extracted using the total RNA isolation kit Nucleospin RNAII (Macherey-Nagel, Duren, Germany). 1 μg RNA was reverse transcribed using the High Capacity cDNA Reverse Transcription Kit (Applied Biosystems, Carlsbard, California, USA). Primers and probes are summarised in a table in the online supplementary section.

A Taqman Low Density Array (TLDA; Applied Biosystems) was used to assess the stability of potential housekeeping genes. Based on the normalisation score, *Cyclophilin A* (*PPIA*) had the lowest variability rate in the samples assayed. Results were normalised using a TaqMan endogenous control (Applied Biosystems).

Diluted cDNA (1:100) was used as a template for the PCR reaction and samples were loaded onto the Applied Biosystems 7900HT Fast Real-Time PCR System. The specificity of the reactions was controlled using ‘no template’ and ‘no reverse transcription’ controls. Results were normalised to the human *PPIA* gene using the standard curve method. Standard curves for the genes of interest were prepared using the plasmids pcDNA3-TLR9-YFP, Addgene plasmid 13642, pcDNA3-TLR4-YFP, Addgene plasmid 13018 and pUC19/human IL-8 Addgene plasmid 17610. Pooled DNA was used in the standard curves for *PPIA*, *TOLLIP* and *TLR2*.

### Immunocytochemistry and confocal microscopy

Confluent cells were detached using trypsin/EDTA solution (10 min at 37°C), and centrifuged. Resuspended cells were seeded onto glass coverslips for 15 min and incubated overnight at 37°C. Medium was replaced with ice-cold methanol for 10 min, the cells were washed and then blocking was performed using 2% goat serum for 30 min. Cells were dried and antibodies were applied overnight as appropriate: murine monoclonal IgG1 against human cytokeratin 18, murine monoclonal IgG2a against human cytokeratin 19, murine monoclonal IgG2a against human TLR2 (all Invitrogen), polyclonal rabbit antihuman TLR4 IgG and polyclonal rabbit antihuman TOLLIP IgG (Abcam). Controls comprised murine isotype monoclonal antibodies (Invitrogen) or, where polyclonal primaries were used, non-immune rabbit IgG (Invitrogen). The following day cells were washed with phosphate buffered saline and secondary antibodies applied for 1 h. Secondary antibodies comprised AlexaFluor488-conjugated goat antimouse IgG (Invitrogen) or goat antirabbit IgG conjugated to AlexaFluor 488 (Invitrogen) as appropriate. Cells were washed, dried and Vectashield with DAPI (Vector Laboratories, Burlingame, California, USA) added. Cells were visualised using a Leica TCS SP5 confocal microscope (Leica Microsystems CMS GmbH, Mannheim, Germany), and photomicrographs taken.

### Coculture of cell lines with *S. aureus*

The cell lines RPMI 2650 or A549 were seeded at a density of 1×10^6^ cells per well. On the same day 5 mL of Modified Eagles Medium (MEM; Sigma-Aldrich) was inoculated with *S. aureus* strain Newman, and incubated overnight at 37°C with continuous shaking. The following day an aliquot was inoculated in 5 mL of MEM and allowed to reach logarithmic phase. Bacteria were washed and resuspended in MEM to achieve an optical density of approximately 0.1. Known volumes were (A) added directly to cells and (B) plated onto tryptic soy agar, so that viable bacterial concentrations could be determined by quantifying colony forming units (CFU) the next day. After infection, cells were incubated for a further 4 h at 37°C prior to cell lysis and RNA extraction as above.

### Statistics

Friedman's test was used to provide a global indication of whether any significant difference existed across the conditions applied to cultured cells. Post hoc analysis comparing unstimulated and stimulated cells was performed using Dunn's test. Comparisons of numerical data between groups were carried out using the Mann-Whitney U test. Comparison of proportions between groups was carried out using Fisher's exact test. Correlations were analysed using Spearman's test. All statistical analyses were performed using GraphPad Prism software (GraphPad Software, La Jolla, California, USA). Statistical significance was considered to be at the p<0.05 level.

## Results

Primary nasal cells were successfully cultured from 6 patients, and primary alveolar cells from 7 (in two cases nasal and alveolar cell were cultured from the same patient). The two groups of patients were similar in their baseline characteristics, although there were more women in the group providing alveolar cells (results from the patients providing nasal cells appear first in all the following comparisons: median age 65 vs 60 years; smoking history100% vs 71%; women 50% vs 86%; mean forced expiratory volume in 1 s 85% vs 84% of predicted; mean diffusing capacity for carbon monoxide (Tco) 63% vs 75% of predicted; no significant difference for any of the comparisons). The patients were admitted for resection of non-small cell lung cancer, with the exception of two patients admitted for resection of solitary metastases. Characterisation by quantitative reverse transcriptase PCR (qRT-PCR) demonstrated that cultured nasal epithelial cells consistently expressed the epithelial cell markers cytokeratin 18 and 19 and alveolar epithelial cells expressed the type II pneumocyte markers SP-C and AQP-3 (data not shown, methods described in the online supplementary section).

A range of bacterial virulence factors was applied to primary cells and the cytokine responses were examined by CBA and qRT-PCR. All of the cytokines examined could be produced by primary nasal epithelial cells. However, none of the measured cytokines were significantly upregulated by exposure to PGN, LTA, LPS or CpG (table 1). In contrast, exposure to TNF induced a significant upregulation of IL-8 and IL-6 secretion (but not the other cytokines studied).

**Table 1:** Constitutive and stimulated cytokine production by primary nasal epithelial cells

		Stimulant				
	Basal	*Staphylococcus aureus* PGN	*S. aureus* LTA	*Pseudomonas aeruginosa* LPS	CpG	TNF
IL-1β (pg/mL)	*7.1* 0–18.7	*7.7* 0–23.8	*4.2* 0–21.9	*3.6* 0–16.4	*6* 0–17.3	*8.1* 0–165
IL-6 (pg/mL)	*29.7* 13.7–313	*140* 21.6–695	*52.1* 6.3–459	*139* 7.9–279	*45* 4.7–535	*956* ** 67.5–3173
IL-8 (pg/mL)	*504* 192–1557	*1363* 378–3821	*663* 297–2309	*740* 131–4295	*520* 11.8–2531	*7817* *** 2033–48 688
IL-10 (pg/mL)	*9.2* 4–18.7	*12.5* 4–21.6	*7.1* 0–14.5	*6.4* 0–18.6	*6.5* 0–21.1	*13* 0–67
IL-12 (pg/mL)	*13.2* 3.6–19.8	*12.1* 0–21	*8.8* 0–16.1	*10.3* 0–21.4	*10.4* 0–26.7	*10.4* 0–23.3
TNF (pg/mL)	*10* 1.7–15	*6.2* 2–24.3	*7.2* 0–11.8	*6.5* 3–16.1	*6.3* 0–17.5	†

Data are expressed as median (upper line, italic) and range (lower line, normal text). n=6 for all conditions. PGN and LTA were applied at 10 μg/mL, LPS at 100 ng/mL, CpG at 1 μM and TNF at 10 ng/mL. Statistical analysis was by Friedman's test and Dunn's post hoc test. *p<0.05, **p<0.01, ***p<0.001 relative to basal levels, by Dunn's post hoc test.

†TNF was used as a positive control; TNF was not measured in TNF-stimulated cells.

IL, interleukin; LPS, lipopolysaccharide; LTA, lipoteichoic acid; TNF, tumour necrosis factor; PGN, peptidoglycan.

Alveolar cell responses were assessed in parallel with nasal cells. LPS and LTA failed to significantly alter secretion of any of the cytokines (table 2). However, in contrast to the nasal cells, exposure to PGN significantly increased production of all cytokines studied in alveolar cells from every patient studied, with the exception of IL-12, suggesting a differential TLR2 response in primary human alveolar versus nasal epithelial cells. Similarly to the response of primary nasal cells, TNF-mediated stimulation induced significant elevations in secretion of IL-6, IL-8 and IL-10 from alveolar cells, suggesting no major differences in signalling downstream of the TNF receptor between these two cell types.

**Table 2:** Constitutive and stimulated cytokine production by primary type II alveolar epithelial cells

		Stimulant				
	Basal	*Staphylococcus aureus* PGN	*S. aureus* LTA	*Pseudomonas aeruginosa* LPS	CpG	TNF
IL-1β (pg/mL)	*5* 2.5–8	*17.2* ** 5–152	*3.4* 1.6–12.5	*6.3* 2.2–14	*7.5* 1.7–11	*11* 1.2–85.3
IL-6 (pg/mL)	*236* 8.3–1276	*927* * 121–9060	*333* 7.6–716	*214* 8.2–533	*228* 12.6–803	*1205* ** 34.1–4029
IL-8 (pg/mL)	*2273* 707–11 226	*7444* * 1283–100 000	*2002* 843–21 914	*1507* 649–13 548	*2919* 636–40 775	*31 721* *** 9450–78 198
IL-10 (pg/mL)	*15* 2.6–1276	*25.4* ** 3.5–5000	*23.2* 3.6–716	*19.2* 3–504	*20.2* 0–803	*26* * 3.5–4029
IL-12 (pg/mL)	*8* 5.4–19.7	*7.3* 6.6–31.2	*8.3* 4.9–30	*12.7* 3.5–25	*12.0* 2.7–28.6	*7* 2.7–30.3
TNF (pg/mL)	*10* 3.6–21.2	*29* * 6.5–279	*5* 0–21.4	*12* 2.3–26.7	*7.0* 0–15.7	†

Data are expressed as median (upper line, italic) and range (lower line, normal text). n=7 for all conditions. PGN and LTA were applied at 10 μg/mL, LPS at 100 ng/mL, CpG at 1 μM and TNF at 10 ng/mL. Statistical analysis was by Friedman's test and Dunn's post hoc test. *p<0.05, **p<0.01, ***p<0.001 relative to basal levels, by Dunn's post hoc test.

†TNF was used as a positive control; TNF was not measured in TNF-stimulated cells.

IL, interleukin; LPS, lipopolysaccharide; LTA, lipoteichoic acid; TNF, tumour necrosis factor; PGN, peptidoglycan.

Given the differential secretion of IL-8 in response to PGN, the effect of this bacterial TLR agonist on IL-8 mRNA production was also analysed. No significant increase in IL-8 expression was observed in either cell type (data not shown), suggesting that at least some of the effect of PGN on IL-8 secretion in alveolar cells may be post-transcriptional.

**Figure 1:**
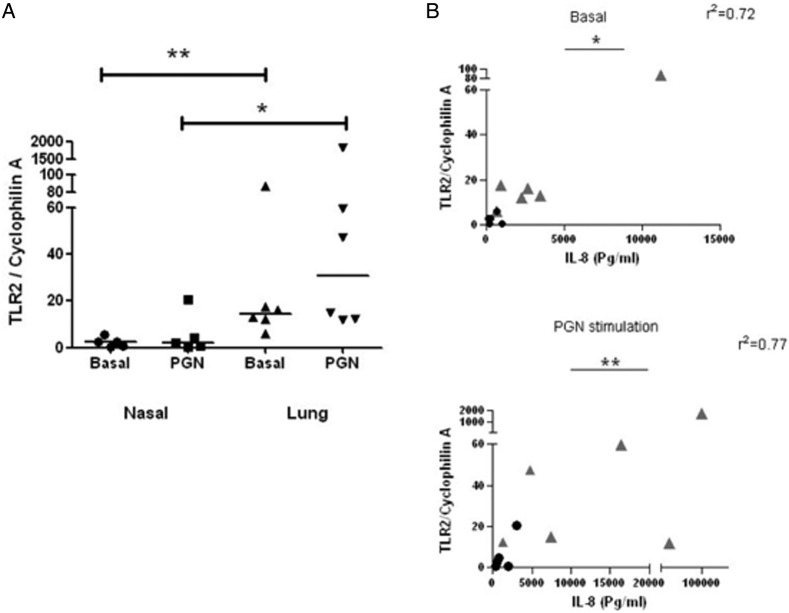
*TLR2* expression is significantly higher in alveolar epithelium than in nasal epithelium, and correlates with IL-8 secretion. (A) Comparison of TLR2 expression in primary nasal and alveolar epithelium, in the presence or absence of PGN. *p<0.05, **p<0.01 using the Mann-Whitney U test. (B) Correlation between TLR2 expression and IL-8 secretion in primary cells, in the presence or absence of PGN. Dots represent nasal epithelial cells, grey triangles represent alveolar cells. *p<0.05, **p<0.01 using Spearman's rank correlation coefficient. TLR, Toll-like receptor; IL, interleukin; PGN, peptidoglycan.

Given that PGN mediates its effects largely through TLR2-mediated recognition and signalling, expression of TLR2 in primary nasal and alveolar epithelial cells was also assessed by qRT-PCR (figure 1A). TLR2 expression was significantly greater in alveolar epithelial cells than in nasal cells (p=0.0043). In contrast, no significant differences in expression of TLR4 and TLR9 were observed between these two cell types (data not shown). Interestingly, TLR2 expression correlated significantly with IL-8 secretion in nasal and epithelial cells, both under basal (p=0.0144) and PGN-stimulated (p=0.0074) conditions (figure 1B).

**Figure 2:**
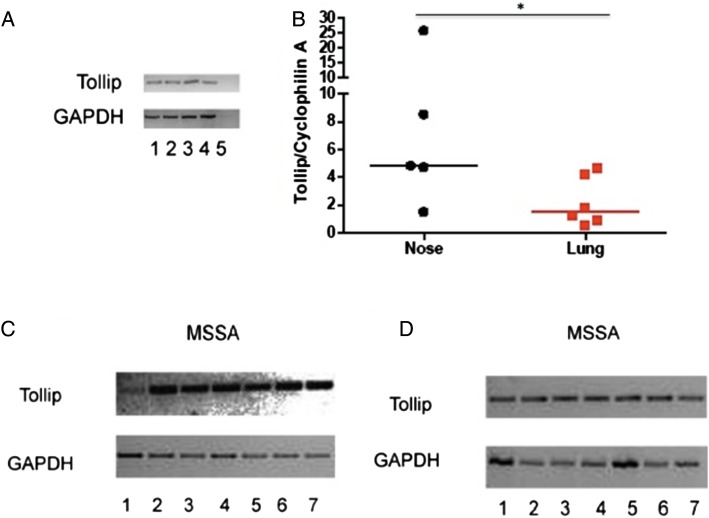
*TOLLIP* expression in nasal and alveolar epithelium. (A) T84 cells were plated at two different cell densities: 5×10^5^ per well (lanes 1, 2); 2×10^6^, (lanes 3, 4). Lane 5 represents a negative control without the reverse transcriptase. *GAPDH* was used as a housekeeping gene. (B) *TOLLIP* expression was quantified in primary nasal and alveolar epithelium. *p<0.05 by Mann-Whitney U test. (C and D) Cell lines were infected with *Staphylococcus aureus* strain Newman. RNA extraction was performed followed by RT-PCR. Panel C shows RPMI 2650 cells—and panel D A549 cells—infected with *S. aureus*. Lanes: (1) positive control for *TOLLIP* from cell line T84; (2 and 3) unstimulated; (4 and 5) cells with *S. aureus* at 1.1×10^5^ cfu/mL; (6 and 7) cells with *S. aureus* at 1.6×10^5^ cfu/mL. *GAPDH* was used as a housekeeping gene. Band size for *TOLLIP* 347 bp and for *GAPDH* 727 bp (TOLLIP, Toll-interacting protein; RT-PCR, reverse transcriptase PCR).

In addition to differential expression of TLR2, the expression of the TLR regulator TOLLIP was evaluated. *TOLLIP* expression has been clearly defined in the T84 colonic carcinoma cell line^6^; therefore, we initially characterised our novel *TOLLIP* qRT-PCR assay in this setting. A band of the expected size was consistently detected, and was absent in negative controls (figure 2A). *TOLLIP* expression was quantified in cultured primary nasal and type II alveolar epithelial cells (from n=5 and n=6, respectively) treated under identical conditions. Basal *TOLLIP* mRNA expression was observed in nasal and alveolar cells but was found to be significantly higher (p<0.05) in the primary nasal epithelial cells (figure 2B).

Owing to the difficulties in obtaining sufficient numbers of primary cells, and the difficulties inherent in applying live bacteria to cells, the effect of *S. aureus* on *TOLLIP* expression was studied in cell lines. Clear evidence for basal *TOLLIP* expression was observed in nasal and alveolar cell lines, and 4 h exposure to *S. aureus* did not appear to influence this (figure 2C, D), suggesting a non-inducible expression in these cell types.

**Figure 3:**
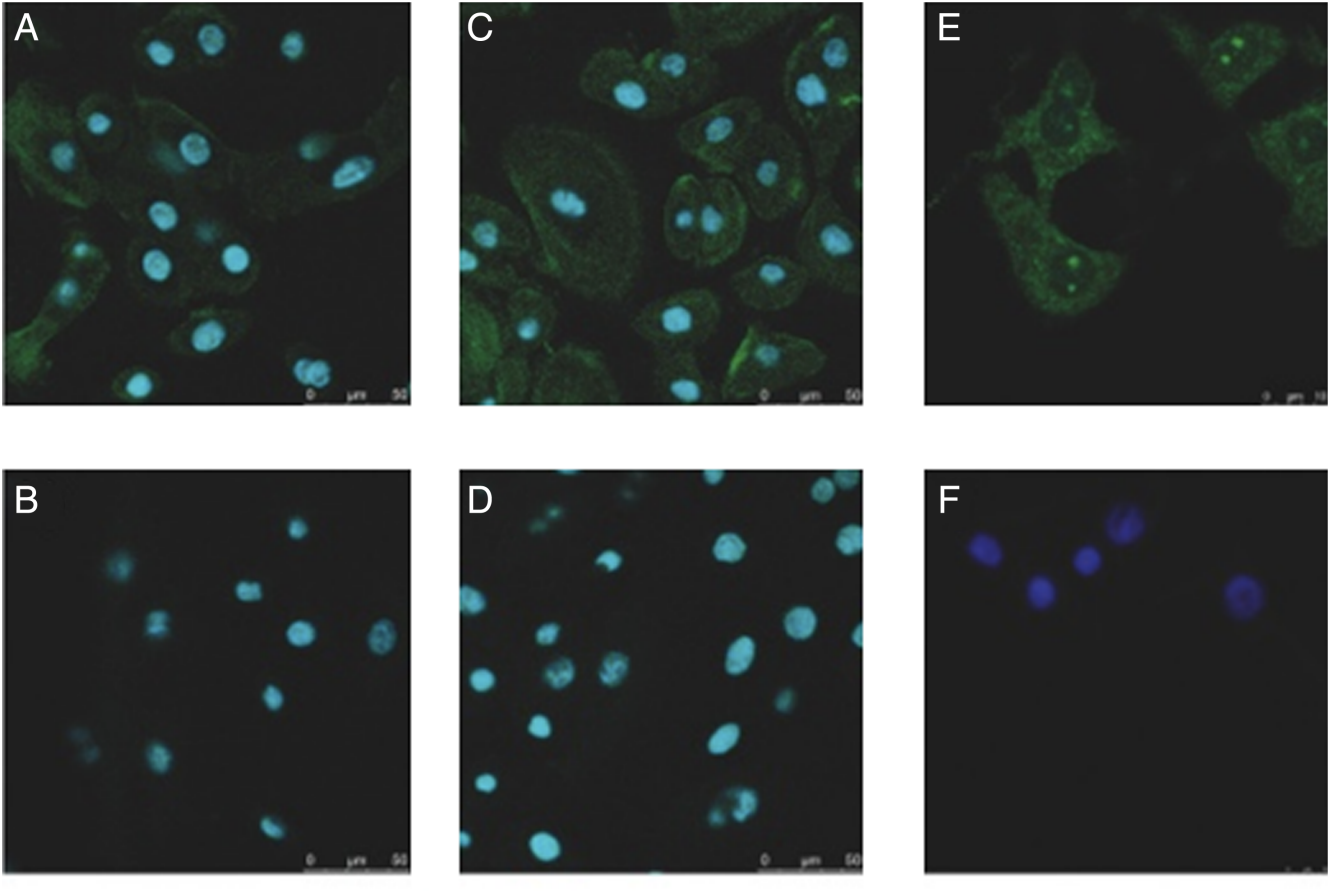
TOLLIP is found in primary human nasal, bronchial and alveolar epithelial cells. Primary nasal (A and B), bronchial (C and D) and type II alveolar epithelial cells (E and F) were fixed, blocked with 2% goat serum and incubated with a rabbit polyclonal antibody against TOLLIP (A, C and E) or isotype control (B, D and F). Nuclei were stained with DAPI (blue). Secondary antibody was antirabbit IgG conjugated with Alexa 488 (green). Images were analysed using confocal microscopy. Three nasal samples, one bronchial and one alveolar were analysed. Scale bar equals 50 μm in A–D, and 10 μm in E and F (TOLLIP, Toll-interacting protein).

Primary nasal and bronchial epithelial cells demonstrated a broadly similar pattern of TOLLIP protein expression, with diffuse punctate staining throughout the cytoplasm, and a suggestion (in a proportion of cells) of peripheral accentuation of staining around the cell membrane (figure 3A–D). Punctate staining was also visible in type II alveolar epithelial cells (figure 3E, F).

## Discussion

To our knowledge, this study is among the first to compare the differential response of primary human nasal and alveolar epithelial cells to a range of identical inflammatory stimuli, and the first to systematically describe TOLLIP expression and localisation in the human respiratory tract.

The findings suggest that primary nasal epithelial cells have a relatively limited repertoire of responsiveness to inflammatory stimuli, generating a statistically significant (but still numerically modest) increase in the proinflammatory cytokines IL-6 and IL-8, only in response to stimulation with TNF, but not TLR agonists. This responsiveness to TNF is consistent with findings elsewhere.^7^ Other studies have suggested that primary human nasal epithelial cells have a relatively restricted nasal cytokine responsiveness to stimulation, broadly in keeping with findings here.^9^ ^10^ However, unlike our results, both these studies found responsiveness of IL-8 to a variety of stimuli, while a further study found that both IL-6 and IL-8 were increased in response to LPS.^11^

In contrast to the relative quiescence of primary nasal cells, we found that primary alveolar epithelial cells were characterised by a more florid response to PGN and TNF that spanned a wider range of cytokines. These observations appear consistent with the hypothesis that bacterial virulence factors are better tolerated by the nose. Our data suggest that *S. aureus* PGN induces a particularly florid inflammatory response in alveolar epithelial cells. It may be particularly relevant that, in our hands, the levels of expression of TLR2 (which recognises PGN) correlated closely with responsiveness, as assessed by IL-8 secretion. The implication seems to be not only that alveolar epithelium expresses more ‘target’ for PGN, but that PGN can upregulate TLR2 expression more effectively on alveolar epithelium. This may go some way to explaining the differential responsiveness of nasal and alveolar epithelium, and perhaps why the lung mounts such a striking inflammatory response to *S. aureus*, a common ‘coloniser’ of the human nose.^12^ It is far less clear why PGN produced a proinflammatory response in our alveolar epithelial cells while LTA and LPS did not. In the case of LPS, the lack of responsiveness could not be attributed to an absence of appropriate receptors, as TLR4 is well described on alveolar epithelial cells, and other groups have described LPS responsiveness in alveolar epithelium.^13^ ^14^ The apparently selective and florid response of alveolar cells to PGN in our hands is intriguing. It is tempting to speculate that membrane-based TLR regulators may recognise different virulence factors preferentially, and/or that PGN effects intracellular TLR regulators in a different way from other virulence factors in primary alveolar epithelial cells. However, this must remain purely speculative until further data are available.

To investigate further potential reasons for differential innate immune responsiveness between the nose and lung, we drew on data describing an excess of TOLLIP in the large intestine, where bacterial tolerance is essential. We believe this to be the first systematic characterisation of TOLLIP's presence and location in primary cells from the human respiratory tract. TOLLIP has been cloned from a human lung cDNA library,^15^ and expression has been described in pooled human lung tissue,^16^ but the purpose of those studies did not include cellular localisation. *TOLLIP* mRNA and TOLLIP protein have been detected in commercially available human small airway epithelial cells.^17^ *TOLLIP* mRNA has also been described in pleural effusions.^18^ Our findings in figure 3 complement those in small airway epithelial cells by suggesting that TOLLIP is produced throughout the length of the human respiratory tract. These observations are at variance with our initial hypothesis. However, the finding of higher *TOLLIP* mRNA expression in primary nasal epithelial cells in comparison to type II alveolar epithelial cells broadly supports the hypothesis. The observation that TOLLIP is constitutively and ubiquitously expressed in human respiratory epithelium is consistent with a potential role as a key regulator of inflammatory responses.^3^ ^4^ ^19^ However, we must stress that we found no evidence for differential *TOLLIP* responsiveness to bacterial virulence factors in nasal and alveolar cell lines.

TOLLIP binds to IL-1 receptor-associated kinase (IRAK-1), preventing proinflammatory signalling. On stimulation of cells with LPS or IL-1, a receptor complex rapidly forms, incorporating TOLLIP bound to IRAK-1. Sufficient phosphorylation of IRAK-1 allows its dissociation from TOLLIP, and proinflammatory signalling (for example, through nuclear factor κ B) rapidly ensues. TOLLIP is therefore well placed to regulate inflammatory processes. TOLLIP's ready availability in organs regularly exposed to bacteria, such as the gut, nose and lung, seems potentially important in this regard. Interestingly, TOLLIP has been implicated in LPS hyporesponsiveness in human monocytes and human primary intestinal epithelial cells.^20^ ^21^

The functional importance of TOLLIP as a regulator of acute inflammation is supported by emerging clinical data. For example, in the Chinese Han population, increased susceptibility to sepsis is conferred by polymorphisms in the *TOLLIP* gene that result in reduced TOLLIP function.^22^ Similarly, functional polymorphisms in a Vietnamese population have been associated with susceptibility to tuberculosis.^23^ In a Caucasian population, *TOLLIP* gene polymorphisms have been weakly associated with increased susceptibility to atopic dermatitis.^24^ Observational data suggest that *TOLLIP* expression is reduced in tissue from coeliac disease and necrotising enterocolitis.^25^ ^26^

While the data here are some way from having direct clinical relevance, validation of a florid alveolar response to PGN in other cohorts might yield avenues for further exploration. In particular, selective administration of anti-TLR2 or specific TLR regulators early in the florid proinflammatory phase of staphylococcal pneumonia seems theoretically attractive in a condition with continued high mortality despite modern antibiotics and supportive care. The association between TLR2 expression and IL-8 secretion in unstimulated and PGN-stimulated cells is potentially relevant in this regard.

Comparison of responses in primary human cells increases the relevance of this study. However, we recognise that there are several potential limitations. First, all of our patients had cancer and most had a long history of smoking, which is known to affect cytokine secretion by epithelial cells throughout the respiratory tract.^27^ ^28^ We cannot exclude the possibility that smoking or systemic effects of patients’ illness may have altered cytokine production or cellular responsiveness. Second, numbers of patients were small, reflecting low availability and technical issues in obtaining cells. While recognising this limitation, we felt that studying primary human cells would be by far the most relevant way to advance this area. Furthermore, consistent effects in studies of this nature help to generate hypotheses for further investigation. Third, as in any model system, we obviously cannot be certain that isolated, cultured epithelial cells behave as they would in their complex native environment. Finally, while epithelial cells are numerically dominant in the nose and alveoli, we cannot exclude the possibility that our stimuli might induce effects in other, less well-represented cells in these regions. Furthermore, in rodents it has been suggested that type I alveolar epithelial cells (notoriously hard to isolate from humans) respond more floridly to inflammatory stimuli than do type II cells.^29^

In summary, primary human alveolar epithelial cells appear to mount a more exuberant inflammatory response to PGN and TNF than do primary human nasal epithelial cells. PGN's effects may relate to the relative abundance and regulation of TLR2 in the upper and lower airway. TOLLIP is produced throughout the human respiratory tract. TOLLIP is expressed in greater levels in nasal cells than in alveolar epithelial cells, but differential TOLLIP expression in nasal and lung cells in response to bacterial virulence factors was not observed. These data suggest that relative expression of TLR2 and TOLLIP might play a role in the tolerant nature of the nasal epithelium to bacteria. Further studies are required to address a range of remaining questions—these include, but are by no means limited to: whether other TLR regulators are differentially expressed (constitutively or inducibly) in nasal versus alveolar epithelium; whether bacterial virulence factors differentially influence TLR regulator expression within alveolar epithelial cells (favouring a proinflammatory effect of PGN but not the other virulence factors measured here) and whether PGN can evade membrane-based TLR regulators on alveolar cells.
